# Partial repigmentation of vitiligo with tofacitinib, without exposure to ultraviolet radiation^☆^^☆☆^

**DOI:** 10.1016/j.abd.2019.08.032

**Published:** 2020-05-05

**Authors:** Melpone Komnitski, Angelo Komnitski, Amilton Komnitski Junior, Caio César Silva de Castro

**Affiliations:** a School of Medicine, Pontifícia Universidade Católica do Paraná, Curitiba, PR, Brazil; b School of Medicine, Universidade do Vale do Itajaí, Itajaí, SC, Brazil; c Private Rheumatology Clinic, Curitiba, PR, Brazil; d Department of Dermatology, Faculdade de Medicina, Pontifícia Universidade Católica do Paraná, Curitiba, PR, Brazil

**Keywords:** Janus kinases, Phototherapy, Vitiligo

## Abstract

Vitiligo is a disease that causes macules and achromic and/or hypochromic patches, which can affect from small areas to the entire tegument. Treatment options are few and are generally ineffective. Recently, some case reports have appeared which show positive results with the use of Janus kinase inhibitors associated with phototherapy. This report details the case of a patient with rheumatoid arthritis associated with vitiligo in treatment for two years, whose condition partially improved initially after eight months of oral tofacitinib at a dose of 5 mg twice a day, without exposure to ultraviolet radiation and with continuous improvement during these two years of treatment.

## Introduction

Vitiligo is a chronic autoimmune disease which affects around 0.5% of the population.^1^ It presents with macules and achromic and/or hypochromic patches on any region of the body. It is a condition that has triggering factors, such as burns and emotional stress, in addition to genetic predisposition. Treatment is performed with topical and systemic corticosteroids, calcineurin inhibitors, and phototherapy with narrow band ultraviolet (UV) B radiation and phototherapy with UVA associated with psoralen. However, these treatments have limited efficacy.^2^ Several articles have shown that blocking the Janus kinase/signal transducers and activators of transcription (JAK-STAT) pathway with tofacitinib may produce repigmentation, provided frequent exposures to the sun or phototherapy.^3^

## Case report

A 40-year-old female patient with comorbid rheumatoid arthritis and vitiligo. Her brother and mother have a positive history for vitiligo. The vitiligo condition started in 2012, with hypochromic and achromic macules in the left inguinal region.

In the same year, she sought medical attention. Phototherapy with narrow band ultraviolet B radiation and systemic corticosteroid mini-pulse was performed. At that time, she reported partial improvement for a few months, but other lesions began to appear on her face, neck, elbows, hands, and feet, diagnosed as common vitiligo. She stopped phototherapy treatment in 2013 because she saw little improvement, and had no dermatological maintenance treatment. In 2014, joint pains in her hands started and she received a diagnosis of rheumatoid arthritis. Some previous treatments were reinstated, such as hydroxychloroquine, deflazacort, and loxoprofen, but they did not lead to remission of the arthritis. In 2017, a new treatment was introduced to treat the rheumatologic condition with only tofacitinib 5 mg, twice a day, which obtained a satisfactory result. Coincidentally, after eight months of medication use, the patient noted improvement of the macules and patches, with formation of several islets of repigmentation in the hands and face, without being exposed to any source of ultraviolet radiation, since the patient uses intense photoprotection with sunscreens, rarely exposes herself to the sun, and did not take any trips to the beach during this time of treatment so as not to exacerbate the vitiligo. After two years, complete repigmentation of the forehead and perilabial macules can be noted, as well as partial repigmentation in the posterior region of the neck and upper chest while the patient is still being treated with tofacitinib.

## Discussion

The pathophysiology of vitiligo has still not been fully elucidated, but it is believed that the IFN-γ and the CD8^+^ T cells play a key role in the destruction of melanocytes.^2^, ^3^, ^4^ Evidence shows that the CD8^+^ T lymphocytes produce IFN-γ, which will express CXCL9 and CXCL10 chemokines by keratinocytes, resulting in the recruitment of more CD8^+^ T lymphocytes, and resulting in the destruction of melanocytes by IFN-γ and perforin/granzyme.^2^

In 2013, tofacitinib was approved by the National Sanitary Surveillance Agency for the treatment of rheumatoid arthritis as it acts as an inhibitor of the JAK kinase family of enzymes, mainly JAK1 and JAK3.^3^, ^5^ As such, it interrupts the production of IFN-α and IFN-γ, and some interleukins such as IL-2 and IL-6, decreasing the inflammatory response.^5^, ^6^ However, it was also seen that it was an alternative for patients with vitiligo.

In 2015, Craiglow et al.^7^ reported the first possible mechanism of action of tofacitinib on vitiligo, which proposed that since the signal transduction of IFN-γ occurs through JAK 1/2, the use of an inhibitor of JAK 1/3 could, in fact, block IFN-γ signalling, which would reduce CXCL10 expression, interrupting the vitiligo activity. Kim et al.,^4^ in a more recent case report, showed that in order to have satisfactory results, sun exposure or phototherapy would be necessary simultaneously with drug treatment.

In a retrospective study of ten vitiligo patients on tofacitinib, Liu et al.^2^ also defended the idea that treatment with the JAK inhibitor would require exposure to light. What was proposed is that for repigmentation to occur, two events need to happen: (1) immunosuppression of inflammation of the skin, which is obtained with the use of tofacitinib and (2) stimulation of melanocytes, either by exposure to sunlight or through narrowband UVB.

**Figure 1:**
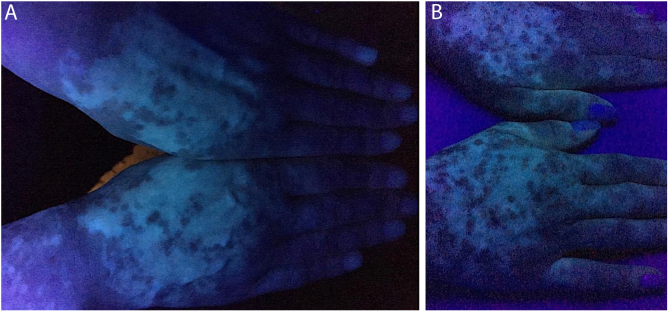
Patient's hands under Wood's lamp. (A) Prior to treatment, various white macules on both hands. (B) After two years of treatment, repigmentation improvement is noted on both hands.

**Figure 2:**
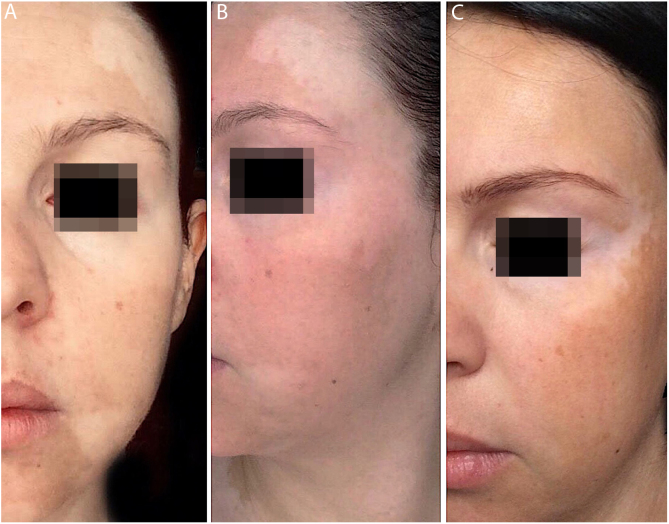
(A and B) Prior to treatment, white macules all over the face. (C) After two years of treatment, complete repigmentation of the forehead and perilabial macules is observed, as well as an improvement of the rest of the face.

**Figure 3:**
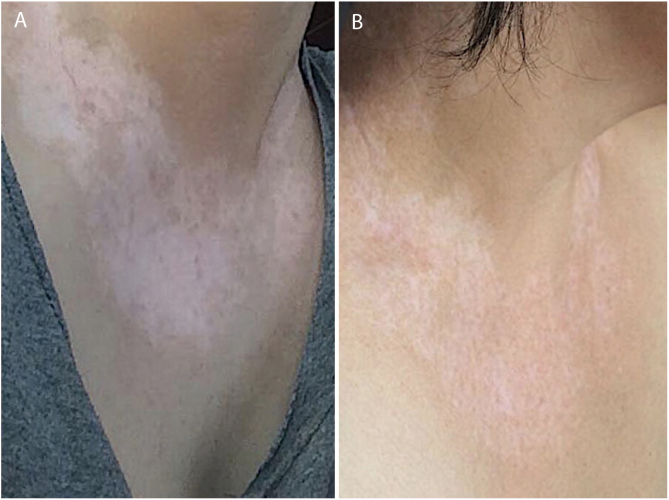
(A) Prior to treatment, various white macules. (B) After two years of treatment, repigmentation improvement is noted.

**Figure 4:**
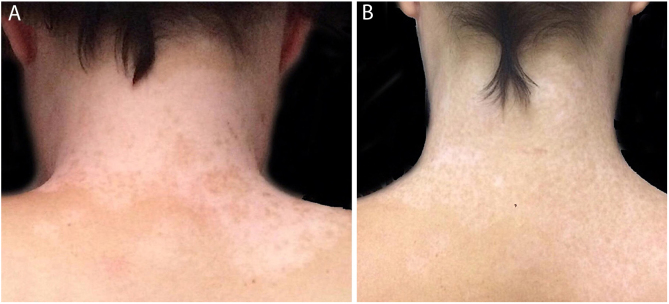
(A) Prior to treatment, numerous white macules. (B) After two years of treatment, repigmentation is nearly complete.

In the case presented, the patient only used 5 mg of tofacitinib twice a day, with improvement in hands (Fig. 1), face (Fig. 2), chest (Fig. 3), and mainly in the cervical region (Fig. 4) where there is no sun exposure. These results are in contrast to the latest case reports, showing that tofacitinib can indeed be effective as monotherapy. It is noteworthy that, unlike the reports and studies on the subject, in which the average treatment time was 5.38 months,^2^, ^4^, ^7^, ^8^ the present patient used the medication long-term, showing that longer treatments will probably be necessary for spontaneous repigmentation to occur after the autoimmune attack ceases, without the patient being exposed to UV radiation to aid in therapy. However, studies of larger populations using tofacitinib who are exposed (or not) to UV radiation are necessary, in order to demonstrate the actual efficacy of this medication.

## Financial support

None declared.

## Authors’ contributions

Melpone Komnitski: Drafting and editing of the manuscript; critical review of the literature.

Angelo Komnitski: Drafting and editing of the manuscript; critical review of the literature.

Amilton Komnitski Junior: Conception and planning of study, drafting and editing of the manuscript; collection, analysis, and interpretation of data; critical review of the literature.

Caio César Silva de Castro: Approval of the final version of the manuscript; conception and planning of study, drafting and editing of the manuscript; collection, analysis, and interpretation of data; participation in design of the study; participation in the propaedeutic and/or therapeutic conduct of the studied cases; critical review of the literature; critical review of the manuscript.

## Conflicts of interest

None declared.
